# Fasting and Post Prandial Pancreatic and Enteroendocrine Hormone Levels in Obese and Non-Obese Participants

**DOI:** 10.1016/j.peptides.2024.171186

**Published:** 2024-03-13

**Authors:** Christopher Bannon, Claire Meek, Frank Reimann, Fiona M Gribble

**Affiliations:** 1 Institute of Metabolic Science, University of Cambridge, Addenbrooke’s Hospital, Cambridge, CB2 0QQ, UK; 2 Cambridge Universities NHS Foundation Trust, Cambridge, CB2 0QQ UK

**Keywords:** Obesity, GLP-1, PYY, GIP, insulin, glucagon, BMI, immunoassay

## Abstract

Circulating insulin levels are known to be increased in people with higher body mass index (BMI) due to effects of adiposity on insulin resistance, whilst gut hormones have a more complex relationship, with fasting peptideYY (PYY) reported to be inversely related to BMI. This study aimed to further explore fasting and post prandial pancreatic and gut hormone concentrations in plasma samples from obese and non-obese participants.

Participants with healthy BMI (n=15), overweight BMI (n=29) and obesity (n=161) had samples taken fasting and 30 min post mixed liquid meal for analysis of glucagon-like peptide-1 (GLP-1), PYY, glucose-dependent insulinotropic polypeptide (GIP), insulin and glucagon. Data visualiation used linear discriminant analysis for dimensionality reduction, to visualise the data and assess scaling of each hormone

Fasting levels of insulin, GIP and PYY were shown to be key classifiers between the 3 groups on ANCOVA analysis, with an observation of increased GIP levels in overweight, but not obese participants. In non-obese subjects, fasting GIP, PYY and insulin correlated with BMI, whereas in obese people only the pancreatic hormones glucagon and insulin correlated with BMI. Concentrations of total GLP-1 in the fasting state correlated strongly with glucagon levels, highlighting potential assay cross-reactivities.

The study, which included a relatively large number of people with severe obesity, supported previous evidence of BMI correlating negatively with fasting PYY and positively with fasting insulin. The observation of increased fasting GIP levels in overweight but not obese participants deserves further validation and mechanistic investigation.

## Graphical abstract

**Graphical Abstract:**
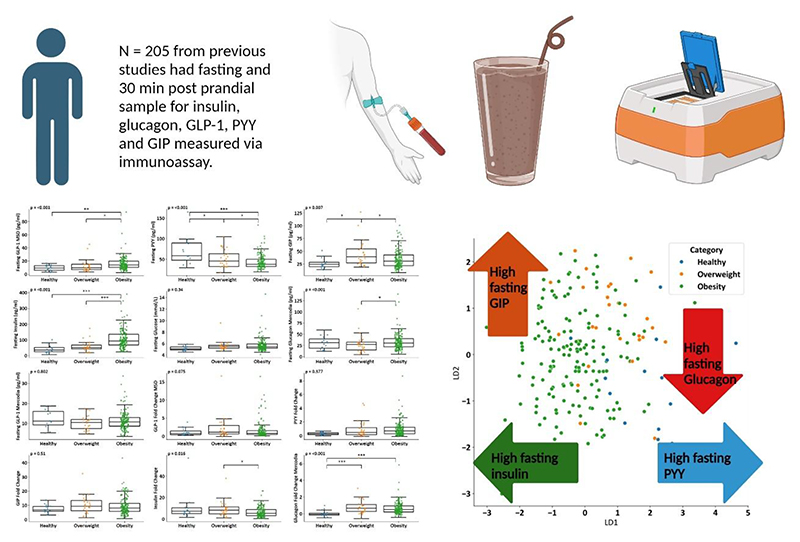

## 1 Introduction

The gut secretes over 20 hormones from enteroendocrine cells, with varying roles including regulation of intestinal motility, glucose homeostasis and appetite [1,2]. Of these hormones, glucagon-like peptide-1 (GLP-1), peptideYY (PYY) and glucose-dependent insulinotropic polypeptide (GIP) have been the most well-studied in humans. GLP-1 and PYY have anorexigenic effects and are key contributors to weight loss post bariatric surgery, increasing 10 fold post operatively in the postprandial state [1]. GIP and GLP-1 are key incretin hormones that modulate release of pancreatic hormones insulin and glucagon to control blood glucose [1,2]. Analogues of GLP-1, alone or in combination with GIP, are used pharmacologically to treat type 2 diabetes and obesity [3].

Fasting and postprandial gut and pancreatic hormones plasma levels are known to vary considerably between individuals. Hyperglucagonaemia and hyperinsulinaemia are well documented in patients with type 2 diabetes and have been extensively reviewed in the literature [4–6]. In addition, fasting insulin levels have been shown to correlate positively with increasing body mass index (BMI) in large cohort studies [7], and fasting hyperglucagonaemia has been observed in obese insulin resistant people without type 2 diabetes [8].

Whilst the mechanisms of disruption to pancreatic alpha and beta cell functioning in obesity due to toxicity of hyperglycaemia and hyperlipidaemia are well explored [9,10], relationships between gut hormones and BMI are less well characterised. Fasting PYY levels have been shown to be inversely related to BMI [11–13], although recent studies have questioned this finding [14], and new analytical methods for PYY using mass spectrometry that distinguish between PYY(1-36) and PYY(3-36) have suggested that PYY concentrations may be lower than previously determined by immunoassay [15,16]. GLP-1 levels post oral glucose were inversely related to BMI, whereas fasting GLP-1 levels were not different between BMI groups in adult participants [17] or slightly reduced in obesity in other studies [18,19]. Increased GLP-1 levels have been reported, however, in children with obesity [20]. GIP levels have shown a diverse pattern in the literature with either increased levels in obese people [21], or no differences in other studies [18,22].

In this study, we analysed plasma hormone responses to a liquid meal test in a group of overweight and obese volunteers and collated these with reference range results from a previous study performed with heathy volunteers [23] to create a database of 205 participants with fasting and 30 mins post prandial GLP-1, PYY, GIP, glucagon and insulin levels. We used this database to explore relationships between BMI and pancreatic and gut hormones.

## 2 Methods

Samples were collected during previously reported studies [23,24]; the reference range data for healthy volunteers were reported previously [23] plasma hormone levels for overweight and obese individuals, whose characteristics are described in Kusinski et al. [24]have not previously been reported. In total samples from 205 participants were available. Studies were approved by research ethics committees (13/EE/0195) [23], (14/WS/0089) [24]

As detailed in publications describing these studies, participants fasted overnight prior to first blood sample, and subsequently consumed a mixed liquid meal comprising 237 ml of Ensure plus containing 11g fat (28%), 13g protein (15%) and 50g carbohydrate (57%) providing 350kcals [23]. Prior diagnosis of pre-diabetes and diabetes (type 1 and type 2) were exclusion criteria for both studies, but a small number of participants were subsequently diagnosed with diabetes incidentally based on their blood tests from the study in which they participated. For this database, fasting and 30 minute post prandial samples were available, comprising total GLP-1, PYY, GIP, insulin and glucagon measurements alongside fasting glucose.

All plasma measurements were performed in the Core Biochemical Assay Laboratory in Addenbrooke’s Hospital. The analytical methods used are given in Table 1. Fasting GLP-1 levels were additionally assayed with the Mercodia total GLP-1 assay in 197 participants for comparison (one outlier GLP-1 Mercodia value with a value above 100pg/ml was excluded from analysis, as the corresponding MSD GLP-1 measurement was not raised). Glucagon was measured using Mercodia’s simultaneous protocol, as the assays were performed prior to recommended protocol change. A proportion of the samples were reanalysed using the sequential glucagon protocol, but the results were not considered reliable and have not been included as a substantial proportion were either below the detection limit, or unexpectedly higher than measured with the simultaneous protocol.

Statistical analysis were performed in R Studio 4.3.3 using the tidyverse, rstatix and emmeans libraries. Data visualisationwere performed in Jupyter Notebook using python 3.9.7 using the packages pandas, numpy, seaborn, matlabplotlib, scipy, pinguoin and scikit-learn.

Post prandial hormone fold changes were calculated for each hormone. For statistical tests, fasting hormone levels were log transformed, and fold changes cube root transformed with normality then checked by histogram inspection. Age and BMI were not transformed in the analysis.

One way ANCOVA tests were performed to compare hormone levels and demographics between BMI categories with age and gender as co-variates, followed by Bonferronipost hoc pairwise comparisons on age adjusted transformed means. For categorical variables, chi squared tests were performed between participant categories.

Correlation heatmaps were plotted for obese and non obese participants, using Pearson’s correlation coefficient in the seaborn package. Correlations are reported either as Pearson’s r or r^2^.

Linear discriminant analysis was performed for dimensionality reduction and data visualisation using the scikit-learn package based upon features selected from one way ANOVA for each hormone feature and BMI category.

Multivariate regression models were built for obese (BMI >30 kg/m^2^) and non obese participants (BMI <30 kg/m^2^) using forward stepwise regression, adding in a feature in order of most to least correlated from heatmap r values, and retaining a feature if in the model its p value remained below 0.1.

## 3 Results

### 3.1 Demographics

On dividing the database of participants into BMI categories, there are clear mismatches in sizes between the groups, with a significantly larger obese group compared to overweight and healthy participants (Table 2). There is also a significant difference in age between groups (p<0.001), with the healthy participants being younger than both the overweight (p=0.028) and obese (p<0.001) cohorts (Supplementary Table 1). Each group had a higher number of female participants, with the sex proportions between groups showing some evidence of difference (p=0.079).

### 3.2 Comparison of total GLP-1 methods

**Figure 1:**
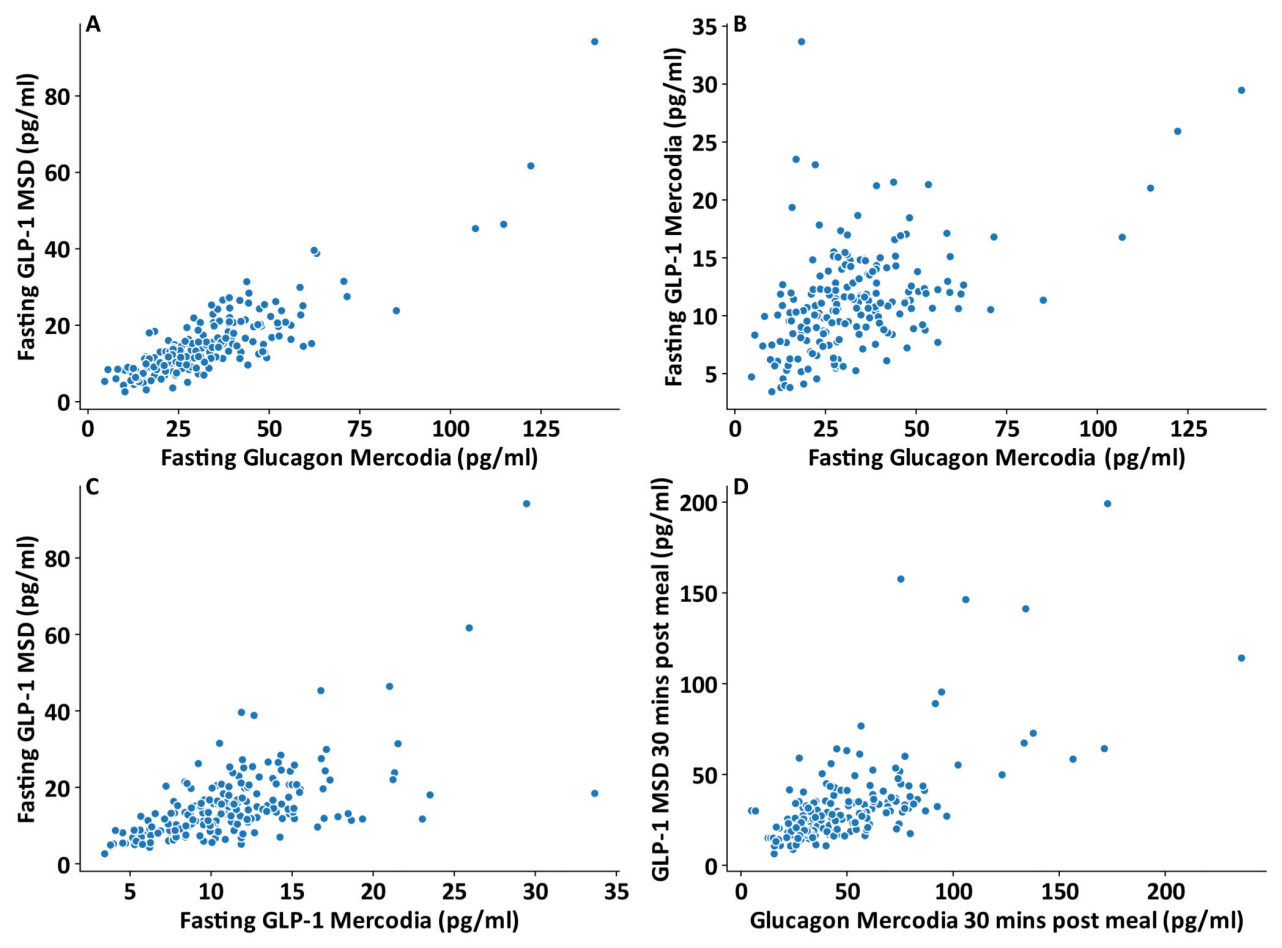
Correlations between total GLP-1 and glucagon measurements A: Scatterplot for fasting total GLP-1 measured with MSD immunoassay vs fasting glucagon measured with Mercodia immunoassay (n = 205, r^2^ = 0.74). B: Scatterplot for fasting total GLP-1 measured with Mercodia immunoassay vs fasting glucagon measured with Mercodia immunoassay (n=197, r^2^ = 0.23) C: Scatterplot for fasting total GLP-1 measured with MSD immunoassay vs fasting total GLP-1 measured with Mercodia immunoassay (n=197, r^2^ = 0.37) D: Scatterplot for 30 min post prandial total GLP-1 measured with MSD immunoassay vs 30 mins post prandial glucagon measured with Mercodia immunoassay (n=205, r^2^ = 0.44) Note in plots B and C one participant was excluded who had a recorded Mercodia GLP-1 result >100 pg/ml.

All participants had a fasting and post prandial MSD total GLP-1 and Mercodia Glucagon measurement, and 198 participants had an additional Mercodia total GLP-1 measurement on their fasting sample. On scatterplots comparing results from the two methods (figure 1), there was strong evidence of association between fasting glucagon and MSD total GLP-1 (r^2^ =0.74), that was reduced when comparing glucagon with Mercodia GLP-1 measurements (r^2^ =0.23). Measurements of total GLP-1 with the two methods showed an intermediate level of association (r^2^ =0.37), as expected, despite systematically lower fasting total GLP-1 levels reported by the Mercodia assay. The correlation between glucagon and MSD total GLP-1 was lower in the 30 minute samples (r^2^ = 0.44).

### 3.3 Fasting hormone levels between BMI categories

**Figure 2:**
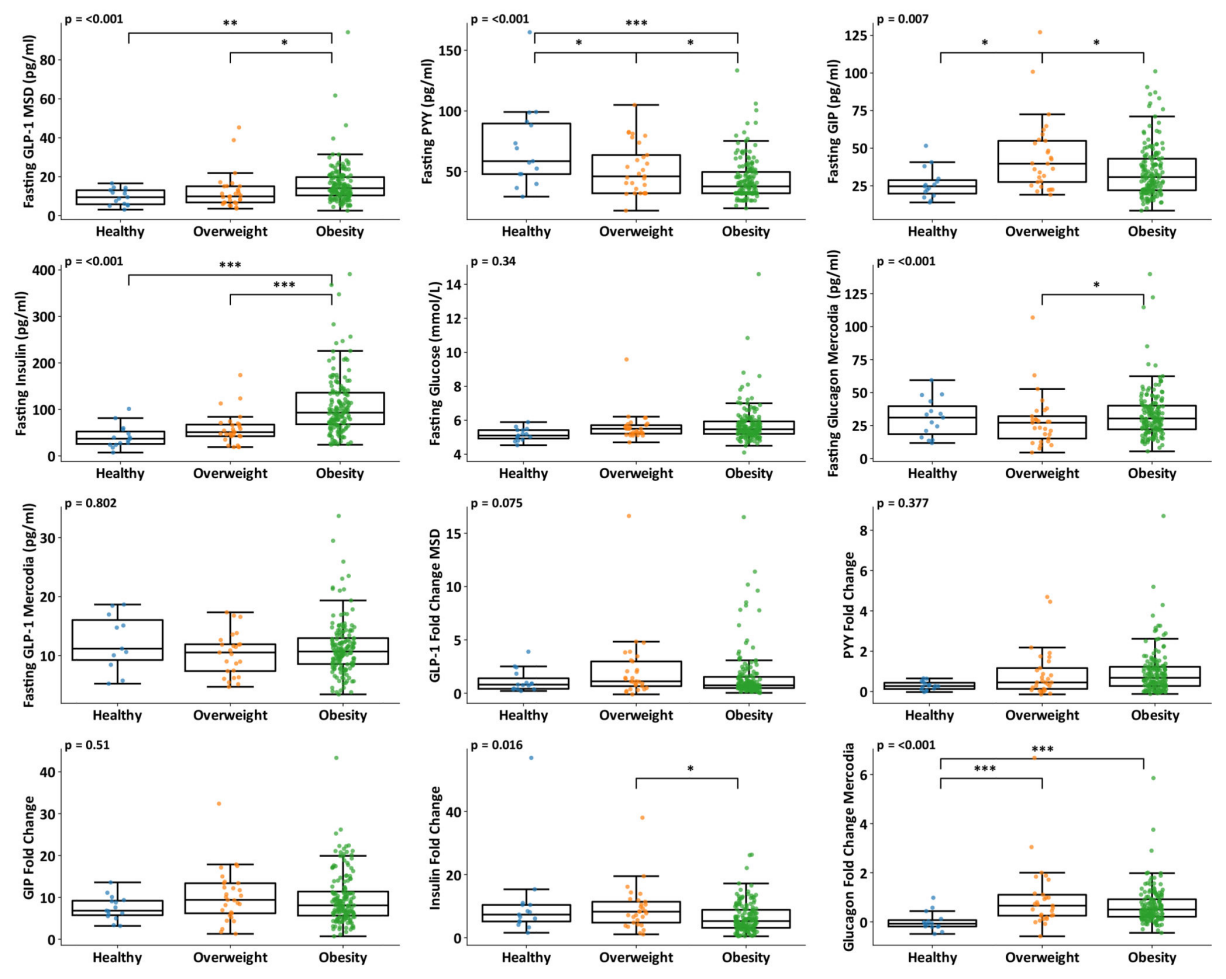
Plasma measurements in lean, overweight and obesity Box plots with individual data points for non-transformed fasting hormone levels, post prandial fold hormone changes and fasting glucose per BMI category. Boxes represent median, IQR and whiskers (lower quartile −(1.5 x IQR), upper quartile +(1.5 x IQR). P values displayed are one-way ANCOVA performed on transformed data, with significance lines for Bonferroni’s pairwise comparison between age adjusted group means on transformed data. Statistical comparisons are detailed in table 2 and supplementary table 1.

On comparing healthy participants (BMI <25 kg/m^2^) with overweight (BMI 25-30 kg/m^2^) and obese (BMI >30 kg/m^2^) participants, fasting insulin (p<0.001), fasting glucagon (p<0.001), PYY (p<0.001) and GIP (p=0.007) all showed significant differences between groups, (figure 2). Whilst fasting GLP-1 levels were significantly different between groups when assessed with the MSD assay, this difference was not apparent when the available samples were analysed with the Mercodia assay.

On pairwise comparison between groups (figure 2 and supplementary table 1), fasting insulin was significantly increased in overweight (p<0.001) and obese (p<0.001) participants compared with the group with healthy BMI. There was very strong evidence of lower fasting PYY levels in the obese group versus participants with a healthy BMI (p<0.001), and significant PYY differences between healthy and overweight (p=0.034), and overweight and obese participants (p=0.047). Fasting GIP was significantly higher in overweight versus healthy BMI participants (p=0.017), but was surprisingly lower in obese compared with overweight participants (p=0.012), with no evidence of a difference in levels between the healthy BMI and obese groups (p=0.817).

**Figure 3:**
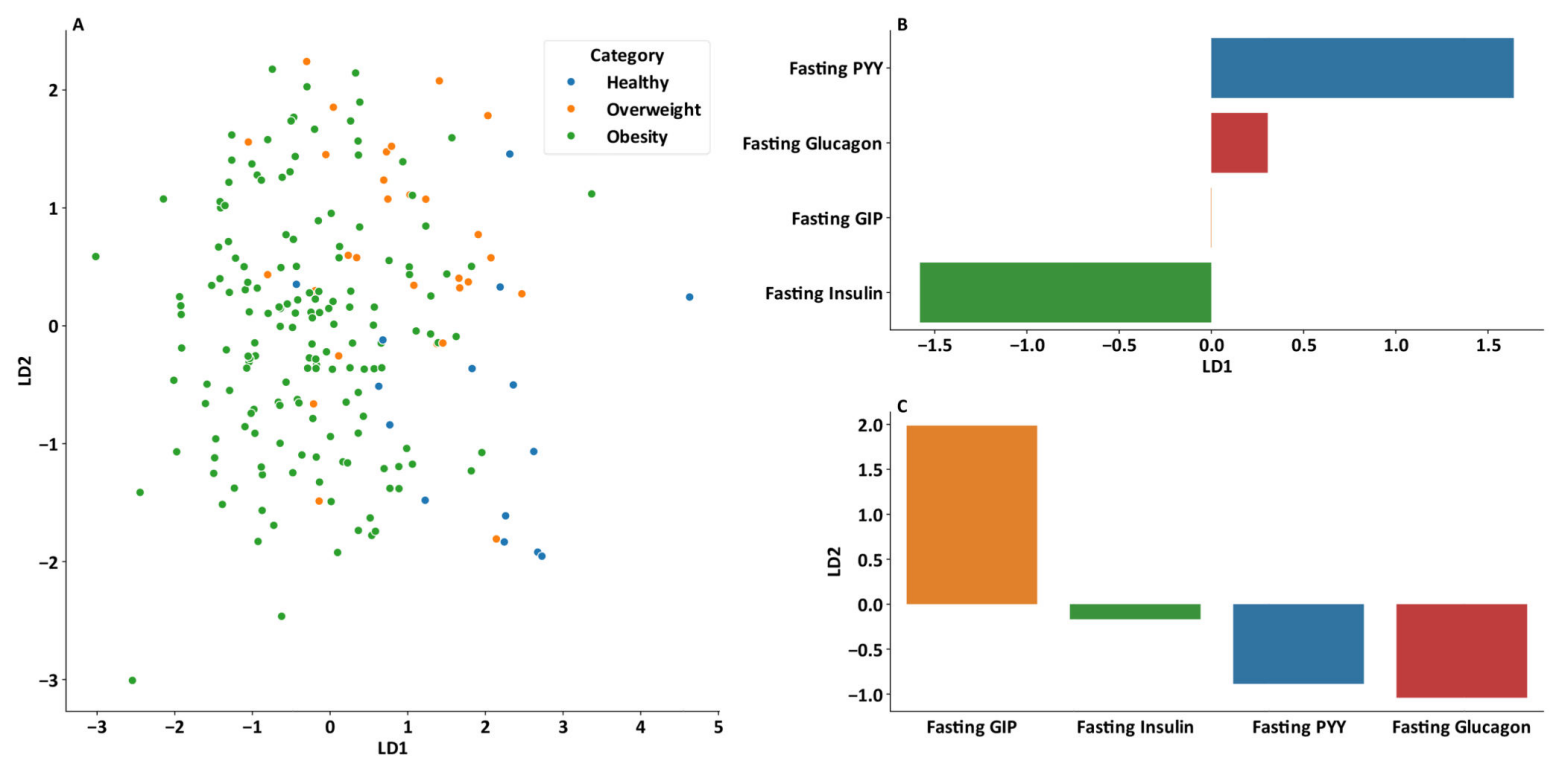
Discriminant analysis based on hormone levels A: Linear Discriminant analysis plot for all participants using transformed fasting PYY, GIP, insulin and glucagon fold change as features. B, C: Scalings for features used for (B) horizontal axis linear discriminant 1 (LD1) and (C) vertical axis linear discriminant analysis 2.

Carrying forward fasting insulin, PYY, GIP and glucagon as selected features, a linear discriminant analysis was performed, and used to plot a visualisation of group separation with scalings for each hormone (figure 3). In this plot healthy BMI volunteers separate towards the right hand side due to higher PYY and lower insulin levels, obese participants towards the left with higher insulin and lower PYY levels, and there was some clustering of overweight participants in the centre upper portion with higher GIP and lower glucagon levels.

### 3.4 Postprandial hormone changes across BMI categories

Fold change in glucagon showed the most significant differences between groups, with higher levels in overweight (p<0.001) and obese participants (p<0.001) compared with healthy BMI participants. The insulin fold change was lower in participants with obesity compared to overweight participants (p=0.042).

### 3.5 Regression models for BMI in obese and non-obese participants

**Figure 4:**
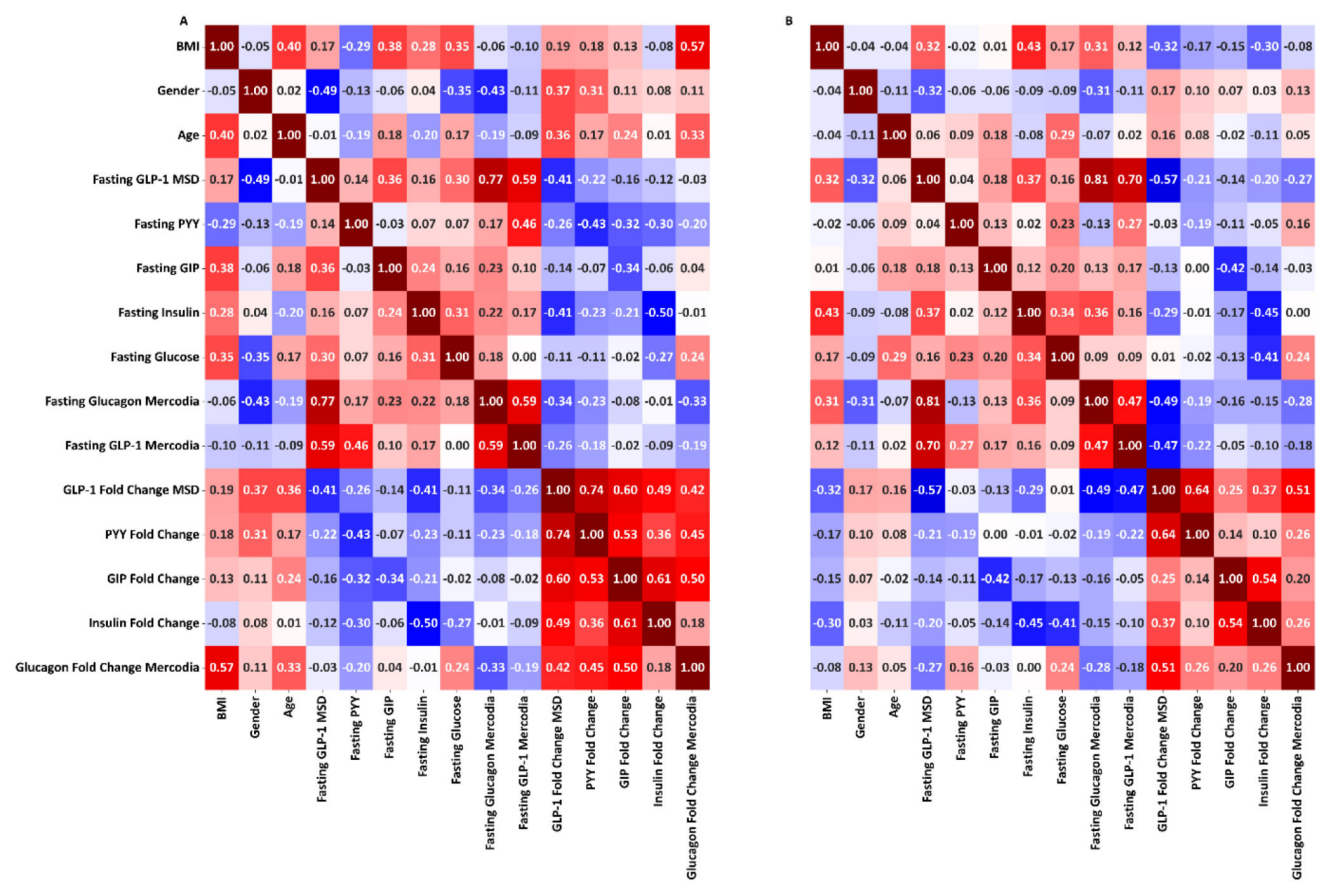
Correlation heat maps A: Correlation heat map for non-obese participants (BMI <30) (n=44; for fasting GLP-1 Mercodia n=36). B: Correlation heatmap for obese participants (BMI >30) (n=161). Correlation coefficients are represented as Pearson’s r.

Correlation heatmaps were produced separately for non-obese (figure 4A) and obese participants (figure 4B) using Pearson’s correlation coefficients on transformed data.

For non-obese participants, BMI showed evidence of positive correlation with fasting GIP (r=0.38), and insulin (r=0.28), and negative correlation with PYY (r=-0.29). Whilst there was a small positive correlation of BMI with fasting MSD GLP-1 (r=0.17), there was no correlation with fasting Mercodia GLP-1 (r=-0.10). Fold changes were all strongly positively correlated with each other, with the glucagon fold change showing the strongest positive correlation with BMI (r=0.57). On further comparing fasting hormones with other features, fasting PYY showed stronger positive correlation with fasting GLP-1 measured using the Mercodia assay (r=0.46) than with the fasting MSD GLP-1 (r=0.14), and no correlation with GIP (r=-0.03), whilst fasting GIP showed stronger positive correlation with MSD GLP-1 (r=0.36) and glucagon (r=0.23) than with Mercodia GLP-1 (r=0.10).

Within the obese group, correlations between BMI and fasting gut hormones were weaker, with no evidence of correlation with fasting PYY (r=-0.02) or GIP (r=0.01). By contrast, the correlation of BMI with fasting glucagon was preserved (r=0.31) and a stronger positive correlation with fasting insulin (r=0.43) was observed. Fasting MSD GLP-1 showed some evidence of correlation with BMI (r=0.32), which was much reduced with the fasting Mercodia GLP-1 measurement (r=0.12). There was modest evidence that BMI in the obese group was negatively correlated with fold hormone changes particularly insulin (r=-0.30), as opposed to the small positive correlations observed with fold hormone changes in non-obese participants. In obese participants, fasting PYY retained its positive correlation with Mercodia Mercodia measurements (r=0.27), and fasting GIP showed weak positive correlations with MSD GLP-1 (r=0.18), Mercodia GLP-1 (r=0.17) and glucagon (r=0.13).

Carrying forward these correlation results, multivariate linear regression models were built in a forward stepwise manner for BMI in obese and non-obese participants, retaining a feature if its p value remained below 0.1. For non-obese patients (table 3), a multivariate model could be built, in which glucagon fold change and fasting GIP, insulin and PYY were able to account for 52% of the variance in BMI. For obese patients (table 4), a multivariate linear regression based on fasting insulin and glucagon and insulin fold change accounted for 22% of the variance in BMI.

## 4 Discussion

In this dataset of 205 participants spanning a wide BMI range, fasting GIP, insulin and PYY were shown to be important classifiers between participants with healthy BMI, overweight and obesity. A combination of these 3 measures, and post prandial fold change in glucagon could account for 52% of the variance in BMI in non-obese participants, whereas in the obese group only pancreatic hormones retained correlation with BMI and only accounted for 22% of the BMI variance.

The correlation between total GLP-1 values generated using the two different assays was surprisingly weak, suggesting different patterns of assay specificity or recovery as highlighted previously as an issue for GLP-1 kits ([25]). As active GLP-1 is formed by proteolytic cleavage from the longer proglucagon peptide, which is differentially processed in different endocrine tissues, there are a number of circulating longer inactive peptides containing the GLP-1 sequence which could cross-react differently with different antibodies. An unexpected strong correlation was observed between fasting MSD total GLP-1 and glucagon measurements, which was weaker for the Mercodia GLP-1 assay. A likely explanation is that the MSD total GLP-1 assay exhibits some cross-reactivity not with glucagon itself but with alternative proglucagon cleavage products generated alongside glucagon in pancreatic α-cells, such as N-terminally extended inactive forms of GLP-1 (GLP-1(1-36amide) and GLP-1(1-37)) and major proglucagon fragment (containing the GLP-1 sequence and GLP-2 in a longer uncleaved peptide). Although pancreatic α-cells produce little active GLP-1 ([26]) unless metabolically stressed, they produce significant amounts of these other longer inactive peptides containing the GLP-1 sequence ([27]) Cross-reactivity of the glucagon assay with glicentin or oxyntomodulin has been reported after gastrectomy surgery [28], but is less likely to contribute to this correlation as levels of these peptides should be low in the fasting state, although were not measured in this study. The findings highlight that caution needs to be taken when interpreting total GLP-1 measurements, particularly in the fasting state when α-cell secretion is high. Other total GLP-1 assays may similarly exhibit cross-reactivity issues, since it is difficult analytically to distinguish gut-derived GLP-1 (starting at the 7-position, or the 9- position after DPP4 degradation) from GLP-1 sequences derived from the pancreas. The positive correlation between fasting Mercodia GLP-1 measurements and PYY is expected, and likely reflects the co-secretion of these hormones from intestinal L-cells.

The observed higher fasting insulin and lower fasting PYY levels in overweight and obese participants support findings from some, but not all, previous studies [7,11,13]. A larger population study did not replicate this association, but measured concentrations of PYY were relatively high in that study. Indeed, recent measurements of PYY(1-36) and PYY(3-36) by mass spectrometry have suggested that total PYY levels may be lower than previously measured by immunoassay (ref the 2 LC-MS papers), questioning which of these methodologies is more accurate. Interestingly, in the current study group, PYY was tightly correlated with BMI across the combined group of healthy BMI and overweight participants, but this relationship was lost at higher BMIs. As the immunoassay measures “total PYY”, our data do not allow us to distinguish which PYY form might drive this association, which is relevant because PYY(1-36) is non-anorectic and insulinotropic, whereas PYY(3-36) is anorectic and not insulinotropic.

The current study highlights an interesting observation that fasting GIP was significantly elevated in overweight participants (BMI 25-30 kg/m^2^) compared with either the obese or healthy BMI groups. Relationships between fasting GIP levels and indices of obesity have been inconsistent in the literature, with some studies reporting higher GIP levels in groups with elevated BMI or waist hip ratio [21], and others finding no significant differences [18,22]. However, group sizes in these studies were relatively small and did not include large numbers of participants with morbid obesity, unlike the current study which included a relatively large group of 161 obese subjects, many with severe obesity (median BMI 39 kg/m^2^; IQR 35-45 kg/m^2^). Interactions between GIP signalling and body weight are complex and incompletely understood. In human population genetic studies, loss of function polymorphisms in GIPR have been associated with lower BMI [29], leading to speculation that GIP signalling can drive body weight gain, and supporting the concept of developing therapeutic GIPR antagonists [30]. By contrast, adding GIPR agonism onto GLP1R agonism in the drug tirzepatide, results in superior efficacy on body weight loss[31], suggesting potential benefits of higher GIP levels. Larger cohort studies including substantial numbers with severe obesity are needed to validate the apparent bell shaped relationship between GIP and BMI observed in the current study, with further mechanistic work required to understand the physiology underpinning this potential relationship and whether it represents direct causality in either direction. A reduction of fasting GIP levels in response to insulin injection was demonstrated in patients with type 1 diabetes [32] which would be compatible with our observation of lower GIP levels in the obese hyperinsulinaemic group, but the proposed negative feedback loop is not widely accepted to be of physiological importance. Obesity associated hypersecretion of GIP is only seen in response to much bigger meals than were used in our study [33].

Whilst changes in fasting insulin and glucagon with BMI may be attributed to insulin resistance [9,10], a strength of this study is the low number of participants with pre-diabetes and type 2 diabetes within the obese groups, further highlighting that hyperinsulinaemia and hyperglycaemia are associated with increasing BMI even without the presence of established diabetes. Enteroendocrine cells turn over every 3-5 days, and should therefore be more resistant against developing chronic metabolic damage than pancreatic α- and β-cells [1,2]. It has been debated whether previously observed low PYY levels in obese subjects are a contributor to or consequence of weight gain [1,11], and whether altered diet such as increased fibre could explain higher PYY in people with lower BMI [1]. Whilst our results support these previous observations, they do not provide a mechanistic explanation.

The main limitation of this study is the big difference in size between our BMI groups, with approximately three quarters of the participants being obese and only 15 participants having a BMI less than 25 kg/m^2^. The healthy volunteers formed a clear cluster on LDA visualisation, and a multi-variate model for BMI findings should be repeated on a larger dataset to further validate these findings. To ensure linear regression models were not over-fitted to patterns of hormone levels in obesity, participants with non-obese and obese BMI were separated in this stage of the analysis, and some different associations were observed in the two groups.

This dataset consisted primarily of female participants, and as such we have made limited comment on any associations of sex with hormone levels. Additionally, the healthy BMI group was disproportionality younger than the other groups, so no reliable observations on relationships between hormone levels and age can be drawn.

## 5 Conclusions

Gut hormones PYY and GIP, and pancreatic hormones insulin and glucagon, as measured by immunoassay, showed strong relationships with BMI in non-obese subjects, whereas in obese people the major BMI correlations were with pancreatic hormones. The observation of raised fasting GIP in overweight but not obese participants requires validation in larger cohort studies, and further mechanistic exploration. We additionally highlight that total GLP-1 assays have potential to cross-react with bio-inactive α-cell products secreted alongside glucagon, which may confuse interpretation of total GLP-1 levels particularly in the fasting state. Further improvements in the specificity and sensitivity of enteropancreatic hormone assays will be crucial for validating the robustness of the associations observed in this and other population studies.

## Supplementary Material

Supplementary Material

## Figures and Tables

**Table 1:** Summary of assays used (18).

Hormone/Analyte	Instrument/Kit	Product code	Supplier
**Glucose**	Siemens’ Dimension	Product code DF40; part number 10444971	Siemens
**Insulin**	DiaSorin Liaison	310360	DiaSorin
**Total GLP-1**	Mesoscale Discovery Total GLP-1 ver. 2 kit	K150JVC-4	MSD
**Total GLP-1**	Mercodia Total GLP- 1 kit	10-1278-01	Mercodia
**PYY**	Mesoscale Discovery Total PYY kit	K151MPD-2	MSD
**GIP**	Mesoscale Discovery Total GIP kit	K151RPD-4	MSD
**Glucagon**	Mercodia Glucagon kit, using simultaneous protocol	10-1271-01	Mercodia

**Table 2:** Table of demographics for participants displayed as median and IQR unless otherwise stated. One way ANOVA or ANCOVA performed on continuous variables and Chi Squared for categorical variables. One way ANOVA performed on non-transformed data for age and BMI; one way ANOCVA performed log transformed data for fasting hormones and glucose and cube root transformed data for fold hormone changes with age and gender as co-variates.

	Healthy N=15	Overweight N=29	Obesity N=161	p-value
**BMI**	22.8 (20.7,24.0)	28.2 (27.3,29.3)	39.4 (35.4,44.8)	<0.001
**Age**	27.0 (23.0,27.5)	34.0 (27.0,45.0)	43.0 (33.0,52.0)	<0.001
**Fasting GLP-1 MSD (pg/ml)**	9.5 (5.8,13.1)	9.9 (6.8,15.1)	14.1 (10.4,19.8)	<0.001
**Fasting PYY (pg/ml)**	58.5 (47.9,89.6)	46.0 (32.0,63.57)	37.7 (32.0,49.6)	<0.001
**Fasting GIP (pg/ml)**	24.7 (19.8,28.8)	39.7 (27.5,54.9)	30.8 (22.0,43.0)	0.007
**Fasting Insulin (pg/ml)**	37.0 (25.7,52.4)	51.0 (42.4,67.4)	93.0 (68.0,136.0)	<0.001
**Fasting Glucose (mmol/L)**	5.1 (4.9,5.4)	5.5 (5.2,5.7)	5.5 (5.2,5.9)	0.340
**Fasting Glucagon Mercodia (pg/ml)**	31.1 (18.6,39.7)	27.2 (15.2,32.1)	30.4 (22.2,40.1)	<0.001
**Fasting GLP-1 Mercodia (pg/ml)**	11.2 (9.3,16.0)	10.5 (7.4,11.9)	10.7 (8.6,13.0)	0.802
**GLP-1 Fold Change MSD**	0.8 (0.41,1.4)	1.1 (0.65,3.0)	0.73 (0.48,1.5)	0.075
**PYY Fold Change**	0.27 (0.13,0.43)	0.45 (0.13,1.2)	0.68 (0.28,1.2)	0.377
**GIP Fold Change**	6.8 (5.7,9.2)	9.4 (6.2,13)	8.1 (5.6,11.4)	0.510
**Insulin Fold Change**	7.3 (5.2,10)	8.3 (4.8,11)	5.3 (3.1,8.9)	0.016
**Glucagon Fold Change Mercodia**	-0.08 (-0.19,0.07)	0.66 (0.25,1.1)	0.5 (0.21,0.92)	<0.001
**Sex**	11 F, 4 M	18 F, 11 M	130 F, 31 M	0.079
**Pre-Diabetes**	0	3	12	0.45
**Type 2 Diabetes**	0	1	5	0.78

**Table 3:** Final multivariate linear regression of BMI predictors from forward stepwise method for non-obese participants, n=44. Overall model: F(4,39), p<0.001, r^2^ = 0.52, adjusted r^2^ = 0.47.

Variable	Coefficient	Std Error	Test Statistic	P value	Coefficient 95% confidence interval
**Glucagon fold change**	2.20	0.48	4.6	<0.001	1.23, 3.17
**Fasting GIP**	1.82	0.70	2.58	0.014	0.39, 3.23
**Fasting PYY**	-1.30	0.77	-1.69	0.10	-2.87, 0.26
**Fasting Insulin**	1.13	0.58	1.96	0.058	-0.04, 2.30
**Constant**	19.7	4.2	4.7	<0.001	11.2, 28.2

**Table 4:** Final multivariate linear regression of BMI predictors from forward stepwise method for obese participants, n=161. Overall model: F(3,157), p<0.001, r^2^ = 0.23, adjusted r^2^ = 0.22.

Variable	Coefficient	Std Error	Test Statistic	P value	Coefficient 95% Confidence interval
**Fasting Insulin**	3.49	0.95	3.66	<0.001	1.60, 5.37
**Fasting Glucagon**	2.38	0.98	2.42	0.017	0.44, 4.32
**Insulin Fold Change**	-2.06	1.16	-1.77	0.080	-4.36, 0.24
**Constant**	20.0	5.7	3.6	0.001	8.9, 31.2
