# Supplementary material for: Fasting and Post Prandial Pancreatic and Enteroendocrine Hormone Levels in Obese and Non-Obese Participants
Source: Peptides. Author manuscript; Available in PMC 2025 Jan 14. (PMC7617300; doi:10.1016/j.peptides.2024.171186)
Supplement: Supplementary Material [file EMS202136-supplement-Supplementary_Material.pdf]

Supplementary Table 1:

Hormone	group1	group2	df	statistic	p	p.adj	p.adj.signif
Fasting_GLP1_MSD	Healthy	Obesity	201	-3.0992	0.0022	0.0067	**
Fasting_GLP1_MSD	Healthy	Overweight	201	-0.899	0.3697	1	ns
Fasting_GLP1_MSD	Obesity	Overweight	201	2.8801	0.0044	0.0132	*
Fasting_PYY	Healthy	Obesity	201	4.6707	0	0	****
Fasting_PYY	Healthy	Overweight	201	2.5511	0.0115	0.0344	*
Fasting_PYY	Obesity	Overweight	201	-2.4399	0.0156	0.0467	*
Fasting_GIP	Healthy	Obesity	201	-1.1014	0.272	0.8161	ns
Fasting_GIP	Healthy	Overweight	201	-2.8046	0.0055	0.0166	*
Fasting_GIP	Obesity	Overweight	201	-2.9251	0.0038	0.0115	*
Fasting_Insulin	Healthy	Obesity	201	-6.6257	0	0	****
Fasting_Insulin	Healthy	Overweight	201	-2.3007	0.0224	0.0673	ns
Fasting_Insulin	Obesity	Overweight	201	5.5555	0	0	****
Fasting_Glucose	Healthy	Obesity	201	-1.3348	0.1834	0.5503	ns
Fasting_Glucose	Healthy	Overweight	201	-1.0462	0.2967	0.8902	ns
Fasting_Glucose	Obesity	Overweight	201	0.1934	0.8469	1	ns
Fasting_Glucagon_Mercodia	Healthy	Obesity	201	-0.92	0.3587	1	ns
Fasting_Glucagon_Mercodia	Healthy	Overweight	201	0.8045	0.422	1	ns
Fasting_Glucagon_Mercodia	Obesity	Overweight	201	2.5574	0.0113	0.0339	*
Fasting_GLP1_Mercodia	Healthy	Obesity	194	0.7731	0.4404	1	ns
Fasting_GLP1_Mercodia	Healthy	Overweight	194	0.6094	0.543	1	ns
Fasting_GLP1_Mercodia	Obesity	Overweight	194	-0.1323	0.8949	1	ns
GLP1_Fold_Change_MSD	Healthy	Obesity	201	0.6296	0.5297	1	ns
GLP1_Fold_Change_MSD	Healthy	Overweight	201	-0.4605	0.6456	1	ns
GLP1_Fold_Change_MSD	Obesity	Overweight	201	-1.607	0.1096	0.3289	ns
PYY_Fold_Change	Healthy	Obesity	201	-1.5376	0.1257	0.3772	ns
PYY_Fold_Change	Healthy	Overweight	201	-0.9208	0.3583	1	ns
PYY_Fold_Change	Obesity	Overweight	201	0.6746	0.5007	1	ns
GIP_Fold_Change	Healthy	Obesity	201	-0.9989	0.3191	0.9572	ns
GIP_Fold_Change	Healthy	Overweight	201	-1.1049	0.2705	0.8116	ns
GIP_Fold_Change	Obesity	Overweight	201	-0.3669	0.7141	1	ns
Insulin_Fold_Change	Healthy	Obesity	201	1.7803	0.0765	0.2296	ns
Insulin_Fold_Change	Healthy	Overweight	201	-0.0003	0.9997	1	ns
Insulin_Fold_Change	Obesity	Overweight	201	-2.4755	0.0141	0.0424	*
Glucagon_Fold_Change_Mercodia	Healthy	Obesity	201	-6.1758	0	0	****

Glucagon_Fold_Change_Mercodia	Healthy	Overweight	201	-5.8035	0	0	****
Glucagon_Fold_Change_Mercodia	Obesity	Overweight	201	-0.6355	0.5258	1	ns

Supplementary Table 1: Bonferroni pairwise comparison p values on age adjusted transformed means. Fasting variables natural log transformed, fold change variables cube root transformed.
